# Review of Domiciliary Newborn-care Practices in Bangladesh

**Published:** 2006-12

**Authors:** Gary L. Darmstadt, Uzma Syed, Zohra Patel, Nazma Kabir

**Affiliations:** ^1^ Department of International Health, Bloomberg School of Public Health, Johns Hopkins University, Baltimore, MD 21205, USA, and Saving Newborn Lives Initiative, Save the Children-USA; ^2^ Washington, DC, USA; ^3^ Save the Children-USA, Dhaka, Bangladesh

**Keywords:** Obstetric care, Behaviour change, Healthcare-seeking, Review literature, Bangladesh

## Abstract

In Bangladesh, high proportions of infant deaths (two-thirds) and deaths among children aged less than five years (38%) occur in the neonatal period. Although most of these deaths occur at home due to preventable causes, little is known about routine domiciliary newborn-care practices and care-seeking for neonatal illness. As an initial step in strategic planning for the implementation of interventions in Bangladesh to improve neonatal outcomes, a review of the literature of antenatal, intrapartum, and postpartum care practices for mothers and newborns in Bangladeshi communities and homes was conducted. A dearth of information was found and summarized, and priority areas for future formative research were identified. The information gained from this review was used for informing development of a guide to formative research on maternal and neonatal care practices in developing-country communities and forms a cornerstone for formulation of behaviour change-communication strategies and messages to advance neonatal health and survival in Bangladesh.

## INTRODUCTION

Each year, approximately four million babies die during the first 28 days of life (i.e. neonatal mortality), and another three million are stillborn (dying between 28 weeks of gestation and birth). Neonatal mortality now accounts for approximately two-thirds of all infant mortality and 38% of deaths of children aged less then five years (under-five mortality) (1). Ninety-nine percent of these deaths occur in middle- and low-income countries with half of deliveries occurring in home (2).

The neonatal mortality rate in Bangladesh is relatively high (41 per 1,000 livebirths in 2003), with most of the estimated 170,000 annual deaths occurring due to infections, birth asphyxia, and complications of prematurity and low birth-weight (LBW) (1, 3). This figure has shown an extremely slow decline over the years. In the time period ranging from 1995 to 1999, the neonatal mortality rate in Bangladesh was 42 per 1,000 livebirths, from 1992 to 1996 it was 48 per 1,000 livebirths, and from 1989 to 1993 it was 52 per 1,000 livebirths (3).

More than 90% of births and neonatal deaths occur at home, generally with little-to-no involvement of the formal healthcare system. Traditional birth attendants (TBAs) attend 75.6%, relatives predominantly attend 10.8%, and medically-trained personnel attend 11.6% of deliveries (4).

Most neonatal deaths can be avoided through simple, affordable interventions, especially in areas with weak health systems and high rates of neonatal mortality. Outreach and family-community care, health education to improve home-care practices, recognition of danger signs, generation of demand for skilled care, and increased health-seeking behaviour can lead to significant reductions in neonatal mortality (5). A programme executed under the five-year Health and Population Sector Programme (HPSP) of the Government of Bangladesh which concentrated on reproductive- and child-health services, limited curative care, and behaviour change communication showed a significant reduction in neonatal mortality from 36.8 per 1,000 livebirths in 1999 to 15.1 per 1,000 livebirths in 2002 (6). Given this context, understanding the domiciliary newborn-care practices and care-seeking for illness are of paramount importance for developing strategies, including behaviour change communications, to prevent these deaths.

Formative research on community newborn-care practices and care-seeking behaviour is required to provide the foundation on which behaviour change-communication programmes can be designed and implemented (7). Such research will need to address the practices of the mother, her nuclear family, TBAs, traditional healers, and formal health providers and facilities. It also must explore the various social, cultural, religious, and economic factors that influence such practices.

The Saving Newborn Lives Initiative (SNLI) of Save the Children-USA undertook a review of maternal and newborn-healthcare practices, specifically: (a) Domiciliary practices: Current maternal and newborn-care practices and behaviours at the community and household levels that directly affect newborn health; (b) Attitudes and beliefs: Behavioural determinants of newborn-care practices, including a review of sociocultural and religious beliefs, and economic determinants behind particular practices; (c) Decision-making: Roles of various individuals (mother, father, mother-in-law, extended family members, neighbours) involved in decision-making about the care of a newborn, including decisions about routine care of a well newborn and decisions relating to the care of a sick newborn. The primary influences, which impact the decision-making process, were also delineated; and (d) Health-seeking behaviour: We sought information on characteristics of those families who elect to use the formal health sector and those who do not. The types of conditions or illnesses that would lead a family to seek care and the type of care they would seek (e.g. formal healthcare sector, traditional healers, private sector) were determined. Various pathways a family uses to seek care for their newborns, and logistical, economic and cultural factors that influence health-seeking behaviour were also considered.

## MATERIALS AND METHODS

An exhaustive literature review of domiciliary maternal and newborn healthcare practices in Bangladesh was conducted. Published manuscripts, reports, and other documents on key areas of maternal and newborn care were identified through searches of all available electronic databases (e.g. Medline, PubMed, Popline). Although most literature cited was specific to Bangladesh, articles from other countries which examined neonatal health and health-seeking behaviour were reviewed to gain a broader perspective on domiciliary newborn-care practices. While several practices have been reviewed and documented, traditional maternal and newborn-care practices within Bangladesh are varied and subject to continuous change over time and geographical region.

Inquiries were made with key-informants from various organizations involved in healthcare delivery in Bangladesh (e.g. local non-governmental organizations [NGOs], such as CARE, Bangladesh Center for Communication Programs [BCCP], BRAC, Institute of Child and Mother Health [ICMH], Bangladesh Institute of Research for Promotion of Essential and Reproductive Health and Technologies [BIRPERHT], Pathfinder, ICDDR,B) regarding their understanding of community knowledge, beliefs, practices, and behaviours relating to newborn care in Bangladesh.

## RESULTS

### Antenatal period

#### Nutrition

Various factors, including cultural beliefs and social status, impact food selections, and, ultimately, nutritional intake of pregnant women in Bangladesh. There is usually more than one decision-maker per household, who could be the father, mother-in-law and/or other relatives, depending on the circumstances (8). A pregnant woman, especially during her first pregnancy, is usually dependent on others and is, thus, in a vulnerable situation, with her well-being impacted most notably by her mother-in-law. Husbands generally lack knowledge of nutritional needs of women, especially during pregnancy and lactation, and defer decisions to their mothers (9, 10).

A family is a highly hierarchical structure in which individuals are differentiated according to kinship roles, which are gender-specific, and which result in unequal rights over resources, including food (9).

Pregnant women and women with children are not involved in fishing or picking green vegetables; it is not customary or prestigious for them to gather these food items (9). It follows in Bangladeshi culture that children and men will be given priority over women for food (9, 11). In this context, pregnancy affords pregnant women no special entitlement to food. The nurturing role of women is pervasive and is expected regardless of circumstances. Women themselves perceive their personal care as dispensable (11). They are expected to take care of others even at the expense of their own health and, as it so happens, the health of the developing foetus.

In general, the nutritional status of pregnant women declines during the food-scarcity period, which is mid-September to mid-November (12). Poor economic conditions of much of the rural and urban population prevent the purchase of nutrient-rich foods (9, 10), but there are no reports regarding the relationship between economic status and purchase of foods or supplementary vitamins and nutrients. The needs of pregnant and lactating women for better quality and larger quantities of food are generally not recognized among either the rich or poor, extended or nuclear families (9).

There are opposing views regarding intake of food during pregnancy that are entertained simultaneously. There is a belief that if a pregnant woman can always keep her stomach full, her baby will not grow too big and she will not face much difficulty during childbirth. Contrary to this is the belief that pregnant woman should eat less, although it is generally acknowledged that pregnant women need more food. Since her abdomen is always full with the foetus, as the argument goes, it is harmful for the baby if she further fills her stomach with food, which thereby limits the space available to the baby and his/her ability to grow. Eating less than her fill will not only leave space for the baby inside to grow but will not hamper her own movements and work due to her excessively large size. The village elders may shame a pregnant woman who eats her fill (9, 13).

An over-riding factor regarding nutrition during pregnancy is the limitations placed on what foods can be consumed and when. A number of studies have documented various restrictions on foods during pregnancy (Table 1) (9, 10, 14–17). The reasons behind the food restrictions are complex, involving aspects of cultural and differential entitlements, taboos, and prescriptions entwined in a myriad of meanings and relationships. Practices vary with localities, but specific sites generally were not specified in the literature. Food intake is sometimes not just a question of availability but also involves fears of eating the wrong food, especially during pregnancy. If a pregnant woman feels sick or has problems with digestion, she reduces her movement and observes a stricter *purdah* (seclusion, avoidance of exposure). In general, hot or spicy foods are avoided during pregnancy. Rice is seen as the essential food for pregnant women. All other foods are considered as side dishes and are relatively less important. Few recognize the nutritional value of vegetables. In some rural areas of Bangladesh, pregnant women consume wheat bread only (18).

**Table 1.** Food restrictions during the antenatal period

Food restricted	To prevent
Protein-rich food	Indigestion
Rickshaw fish	Skin diseases
Prawns	
Coconut	Blindness
Green coconut water (observed by Hindu women)	Baby's eyes being white and glossy
Pineapple, *boal* fish, flat peas	Abortion
Cow's milk	
Cucumber	
Hilsa	
Sour foods	Excessive retention of body fluids
Bananas	Pneumonia
Spinach	
Onion	
Seafood	
Duck	Foetal death

According to the Bangladesh Demographic and Health Survey (BDHS) 2004, only 44% of women recalled being weighed, and 50% recalled receiving iron tablets or syrup, at the time of receiving antenatal care (19). Direct information on reasons for the lack of importance placed on iron and folic acid in the diet of women in rural Bangladesh is lacking.

#### Infections

Knowledge and practice regarding prevention of tetanus through immunization is generally good, due to informational and well-coordinated tetanus toxoid vaccination campaigns (20). In one study, of the 30% of women who attended antenatal care, 83% received tetanus toxoid (TT) vaccination during their visits (15).

Current knowledge, attitudes, and practices (KAPs) concerning HIV/AIDS, other sexually transmitted infections (STIs), and reproductive tract infections (RTIs) are very poor. In general, women and men do not consider themselves nor newborns at risk from STIs or RTIs and are not familiar with the signs and symptoms of these infections. Little information is available regarding common beliefs about the transmission of diseases, including RTIs, from mother to newborn (21, 22).

#### Antenatal healthcare for the mother

About 56% of pregnant women reported visiting a provider for at least one antenatal care check-up, of which only 49% received care from a medically-trained provider (doctor, nurse, or midwife) (23). Seventy-five percent of urban pregnant women had antenatal care from a medically-qualified provider compared to 51% of pregnant women in rural areas (19). Visits for antenatal care were also found by another researcher to be particularly uncommon in rural areas (15). It was noted that 70% of pregnant women did not seek an antenatal care, yet 72% of respondents knew about the location of facilities that provide pregnancy-related care. Few women attended governmental health facilities in one study (23).

Women tend to seek antenatal care late in their pregnancy. The median duration of pregnancy at first visit is 5.2 months among those who did seek care. Antenatal care is more common among younger women and women of lower birth-order. The use of antenatal care is strongly associated with the level of education of the mother and household economic status. The most frequently cited reasons for not seeking antenatal care were lack of need where almost two-thirds of mothers stated that antenatal care provided no benefit. Monetary constraints, not knowing service was needed, religious constraints, restrictions on the movement of women, and low-perceived quality of care were also cited as reasons for not accessing care (19).

In general, it appears that the role of antenatal care in preventing problems during pregnancy and childbirth has not been incorporated into women's belief systems (16). Women who had received antenatal care from trained providers were more likely than those who did not seek antenatal care to have knowledge of the availability of healthcare facilities for their sick newborns. Rahman *et al.* have also found that previous visits to the Union Health and Family Welfare Centres (UHFWCs) and Satellite Clinics were associated with greater seeking for antenatal care services. In some cases, women received antenatal care at home by a health provider, or they had no health problem and so did not think they needed to visit a health centre for routine antenatal care (24).

Like most decisions regarding pregnancy, the mother-in-law usually makes those pertaining to whether and where her daughter-in-law will attend antenatal care for the first baby/pregnancy. Also, the husband and mother-in-law make decisions about the number of children the family should have (9, 10, 25).

Since *dais* (trained or untrained birth attendants) are called only at the onset of labour, it is not customary for them to perform antenatal check-ups, nor to examine the position of the baby in the weeks preceding the birth. If no complications are present, TBAs do not refer the mother for antenatal care, as they feel it is not needed or mandatory (9, 10).

The harvesting and rice-processing season is a period when workload for women is increased considerably. In one study in rural Bangladesh, 63% of pregnant women did not get enough rest, of which 72% reported that this was predominantly due to time constraints. Women who have the highest workload are not the poorest or the ones in the higher economic spectrum but the ones who are slightly above the poorest and have access to resources and employment. They are excessively burdened by household chores and outside labour, which is considered by them as their ‘duty’ or ‘responsibility’ and are much less likely to consider ‘self-care’ in the same terms. Thus, they are more likely to sacrifice personal care to fulfill their duties to their family and generally sacrifice sleep and rest to do so (11).

Little research has been done on the issues of workload, exposure to smoke, and other pollutants, such as lead, and smoking, alcohol, and other drug abuses in pregnant women in Bangladesh.

#### Birth-preparedness

Pregnancy is regarded as a fragile, vulnerable state, yet childbirth is considered a natural event with social and religious ramifications, not a medical event. Women work hard up to the time of delivery and give birth at home or outside the home, stoically and without embarrassing preparations. In this context, there is general reluctance to either prepare for the delivery or to seek care for danger signs during pregnancy or delivery. There is a belief that the more people hear the news about an impending delivery, the more delay of the delivery that will occur. In the Bangladesh Maternal Health Services and Maternal Mortality Survey 2001, more than half of pregnant women in their third trimester had still not decided on delivery assistance (26). Thus, birth-preparedness is avoided and is considered embarrassing.

#### Danger signs and complications

Women seem to recognize many complications of labour, e.g. eclampsia/pre-eclampsia, but do not see them as life-threatening. As per the BDHS 2004, 52% of women identified prolonged or obstructed labour as potentially life-threatening, 42% identified retained placenta, 28% identified convulsions, 33% identified abnormal foetal presentation, and only 28% identified excessive bleeding as potentially life-threatening conditions (19). Thus, awareness of life-threatening conditions during pregnancy and during and after delivery is low among Bangladeshi women.

Although families may recognize danger signs, they seem to not associate complications of pregnancy with danger signs (e.g. they do not associate swollen face, hands, and feet with convulsions due to eclampsia) and are quite reluctant to go to a doctor when they recognize a complication or danger sign (14, 15). In one study, 48% of those with reported bleeding did not seek any care, and 22% sought help from unqualified village doctors. Non-formal care is seen as an important source of maternal care (8, 27). In another report, while knowledge of TBAs on complications during pregnancy and the postpartum period was high, few (12 of 50) referred their patients (28).

In the BDHS 2004, 29% of women with one or more life-threatening complications sought care from a medically-trained provider, and one-third sought care from an unqualified provider. Overall, 38% did not seek any treatment for maternal complications around delivery. Treatment-seeking from medically-trained providers varied by type of complication, i.e. it was highest for convulsions (41%) and lowest for excessive bleeding (27%) (19).

TBAs are trained to identify risk factors and refer complicated pregnancies to the hospital. In general, it appears that TBAs understand some-to-many danger signs (14, 15). Although TBAs are trained to identify high-risk pregnancies, they often will try to manage the problem until the last moment and refer to a health facility only when it appears hopeless and the mother is near death (16, 17). If a mother becomes unconscious due to eclampsia, efforts typically will continue at home, as spoons are forcefully stuck into the mouth to open it (14, 15). Hospitals are considered as the last resort (14, 15), and few women with complications are either referred or taken to formal health facilities for care. Many perceived dangers during pregnancy are thought to come in the form of wild spirits who may possess a woman; for example, if she falls or ventures outside and exposes herself at inappropriate times and places (9, 10).

Unqualified village doctors typically are consulted for remedies. Prolonged labour is perceived as serious, and in one study, care was sought from mostly unqualified village doctors (29). Excessive bleeding or fits/convulsions and fever for more than three days, shock/loss of consciousness, and foul vaginal discharge are also considered serious by these providers (14, 15). Convulsions and fever are thought to be life-threatening during pregnancy, although fits/convulsions after delivery are not taken seriously by untrained TBAs, who do not bother to refer them to or summon a doctor. Eclampsia and spasms of tetanus are not recognized as separate diseases. Tetanus is believed to be a spell of the evil spirit (9, 10), and eclampsia/pre-eclampsia is treated by beating the mother with a broom stick or placing burnt chillies at her nostrils to smell, as it is believed that the condition is the spell of a spirit and doing so will get rid of it (14, 15). Eclampsia, infection, and haemorrhage—the major causes of maternal mortality—may be exacerbated by traditional Islamic birthing rituals (13).

For life-threatening complications, the husband was the primary decision-maker concerning seeking treatment, although other important decision-makers included the woman's parents and parents-in-law. The prominence of the woman's parents in decision-making may reflect to some extent the practice of returning to the natal home for the woman's first delivery (26).

### Intrapartum period

About one-third of deliveries in Bangladesh are unassisted, and for many of the remainder, help is provided from relatives (considered *dais*) or practising *dais* (considered TBAs). The term *dai* can refer to either category of delivery attendant. They are the most common birth attendants and the first to be called, particularly in rural areas. *Dais* are loosely defined by community standards of experience or training, but proper social status is of vital importance in qualifying to be a *dai*. *Dais* are specialists at delivering babies without use of instruments or medications. They have practical knowledge of a woman's anatomy, and many use their hands to manipulate the baby's position the birth canal when necessary to facilitate delivery and can handle delayed delivery of the placenta. Since the *dai* usually is the only woman in a chain of possible delivery attendants, some stress the importance of their role in preserving women's honour, i.e. preventing women from being delivered and, thus, seen by a man. Guardians encourage the *dai* to persist in a difficult case, so as to avoid doctor's fees, the frightening experience of the unknown city and the dishonour of a wife being attended by male doctors (9, 10).

Difficulties in childbirth that are attributed to spiritual agents are usually intervened by a different kind of specialist, a *mowlana* or a *fakir* (a spiritual man). The latter is recognized to have greater spiritual powers and are men. For powerful medicines or instruments, help is sought from various categories of doctors who typically are also men (9, 10).

Data from one study clearly showed that the most significant factor in determining the use of modern healthcare resources was delivery-related complications, followed by paternal education and prenatal education and care (30).

#### Delivery

More than 90% of deliveries in Bangladesh take place at home; of these, one-fourth, typically the woman's first delivery, occur at the parent's home (paternal or maternal). Untrained TBAs conducted 88% of these home-deliveries, while only 9% were performed by trained TBAs (14, 15). Results of a study in urban slums of Dhaka showed that an untrained attendant performed 75% of deliveries, and 8% of women delivered the infant by themselves. Qualified doctors delivered only 3% of babies (31).

**Hygiene:** Knowledge of trained TBAs is high on hygienic delivery practices, including washing hands with soap and water, changing clothes before delivery, and using a new or sterilized blade to cut the cord (28). Trained TBAs reported several events or stages at which they wash their hands, including ‘before holding the baby’ (89.5%), ‘before cutting the cord’ (49.1%), and ‘after delivery’ (94.7%) (14). Yet, TBAs may perform repeated vaginal examinations (e.g. up to 40 per pregnancy), typically with unwashed hands, to determine if it is time for delivery (16).

In general, the hygienic practices of TBAs are not what is expected. Right from preparing a room for delivery, few attempts are taken to keep the process clean and hygienic. Rather than emphasizing the cleanliness of the birth process, concerns over preventing contamination of the household often take precedence. Much emphasis is placed on controlling the pollution of birth, i.e. the fluids and placenta (9, 10). Usually the inside of the home is considered cleaner than the outside. Nevertheless, mothers are taken to a dark room, and on most occasions, the room is ill ventilated. Sometimes, pregnant women are placed beside the room where domestic animals are kept (9, 10, 14–16). During delivery, an unclean polythene plastic sheet and *pati* and/or jute bags generally are placed under the mother. The mother is situated on the earthen floor in the cold season, the cord-cutting utensils (blades) may be placed, for example, on banana tree leaves, dirty rags are used as a mat, and clay is used for stopping bleeding. The mother wears dirty clothes because the birth process is considered unclean (10).

While one of the first lessons taught to *dais* during their training is the importance of washing hands with soap and water and of wearing clean clothes; traditionally, *dais* wash scrupulously, not before, but after the birth. Their notion of dirt or pollution appears unrelated to the germ theory. Women use soap to end the period of pollution, i.e. at the end of menstruation and when they leave the house of seclusion after giving birth. The concept of washing with soap as a cleansing act before delivery to protect against infection is not appreciated nor is the concept of hand-washing with soap applied to the postpartum care of the newborn during the period of seclusion (9, 10).

**Practices to speed delivery of the baby:** Abdominal massage is routine during labour. One-third of all women received abdominal massage as an intervention for accelerating labour to keep the foetus in position and/or to reduce labour pain (14, 15). TBAs sometimes applying force to aid in delivery of the baby and may be excessive, including standing up on or pushing the mother's abdomen with their knees, placing bands around the abdomen, or shaking the abdomen of the mother with the belief that shaking will root out the baby inside (16, 17). TBAs sometimes kick the mother's waist to speed up the delivery and may kick the baby, believing it will protect the baby from evil spirits. Introduction of various substances, e.g. juices of *chalta* fruit, mustard oil, coconut oil, soap-water, and juice of tree buckle, into the vagina to make it slippery and facilitate delivery is common, as is pulling on the umbilical cord (14–17). A Mariam flower may be placed in water (14, 15), and people around the delivery may be asked to untie their hair to speed up the delivery process. Some also believe that, if tidal water from the sea is given to a pregnant woman, it will ease her delivery. Amulets are tied on the thighs of pregnant women to speed up the delivery, and also in some cases of extremely long labour, snails are broken over the pregnant mother's head to cause more rapid delivery. Mothers are asked to do repeated stand-ups and sit-downs. To speed delivery, women may be advised to put mustard oil on their heads and to wear a *saree* reversed. The birth attendant's hands, mustard oil, and garlic are also put in the mouth to make the mother vomit. Molasses juice is given to mothers to eat, and hair, rags, and other items, even kerosene oil in extreme cases, are put into the mouth of the mother to induce vomiting and speed up the delivery. Some are given water that has been used for washing the legs of the father-in-law in the case of prolonged delivery. The mother-in-law may ask the mother to blow into a bottle and then hair is put inside the mouth to induce vomiting. Sometimes, mothers are forced to eat uncooked fresh duck eggs to reduce their labour pain. Walking during labour, squatting for delivery, and not interfering with the membranes (i.e. not breaking them early) could be considered good traditional practices. One reason TBAs usually conduct the delivery with the mother in a squatting position is so that others cannot see the birth passage (9, 10).

**Practices to speed delivery of the placenta:** The focus of attention after birth of the baby is on the delivery of the placenta, as the placenta is believed to have spiritual value, and until then the baby is typically left completely unattended. There is usually a panic if the placenta is not ejected quickly, as the mother is believed to be in danger. Many believe that the placenta can grow inside the abdomen or move up into the throat and choke the woman to death if not removed promptly (16, 17). To release the placenta, rice in a bamboo pot is thrown on top of the roof of the house, and palm trees are shaken. If the placenta is delayed in coming out, *dais*, pressed by other women present, tend to intervene quickly. They do not hesitate to remove it manually (9, 10). After its delivery, the TBA carries out forceful abdominal massages to bring out the clotted blood from the uterus (16, 17).

#### Resuscitation

The placenta is considered the baby's source of life, and, thus, it is delivered before the cord is cut. Treatment of the placenta is usually considered a higher priority than treatment of the newborn immediately after birth. Once ejected, if the baby shows no signs of life, the placenta will be manipulated, heated up, and trampled upon, and the cord will be squeezed and massaged to bring a flow of life from the placenta to the baby. If the baby is not breathing at delivery, it is common to wait to intervene until the placenta is delivered, then while keeping the placenta attached to the baby, it is heated and fried, believing that the baby will get the heat and energy to cry (14, 15). On some occasions, water is poured on the baby's cord to make the baby cry. If the baby does not cry at birth, TBAs also blow into the mouth of the baby, strike the baby heavily on the back or heels, and/or hold the baby upside down. They also pour water onto the umbilicus of the baby and bathe the baby (15).

#### Umbilical cord care

A harmful practice in cord care is the use of a contaminated razor blade, bamboo slice, or other instruments to cut the umbilical cord and the application of potentially infectious material to the umbilical cord stump (3, 4). Some are unaware that unhygienic practices could lead to infection (32). Warm or hot water is used for cleaning the instruments for cutting the cord; sterilization in boiling water typically is not done. In most cases, the cord is cut and tied after the placenta has been delivered, for the reasons noted above (see the section on Practices to speed delivery of the placenta). One study reported that, in 95% of cases, the umbilical cord was cut with a razor blade, 5% used a strip of bamboo, 13% boiled the razor blade for sterilization, and 71% applied nothing to the umbilical cord after cutting (31).

The cord often is tied with dirty thread, such as thread used for sewing quilts or jute fibre (14, 15). The tie typically is placed four finger widths away from the proximal end of the umbilicus. Little attention is paid to tying the cord, however; sometimes, the tie is loose with one knot or with no ties at all (33), which may result in bleeding. Earth of the local oven (*chula*), ashes, lamp soot, powder, dry cow-dung, vermilion (practised by Hindus), or oil may be put on the umbilicus. The umbilicus is sometimes heated to make it dry. Blood of the cord often is put into the baby's mouth and spread over the chest and back with the belief that this will increase bonding with the mother (15).

TBAs do not cut the cord because of the belief that whoever does so remains unholy and cannot go to prayer for 41 days. Most of the time, the mother of the baby has to cut the cord, as she already is considered unclean after the delivery and will be placed in seclusion regardless of whether she cuts the cord. On occasion, a young child who has not yet begun to pray will be given the duty of cutting the cord (10).

#### Hypothermia

Hypothermia is not recognized as a separate entity but, rather, may be considered a manifestation of sickness in a child. Risk for hypothermia may stem from deeply-rooted traditional beliefs and practices. The newborn typically is placed wet and unattended on the ground until after the placenta is delivered. Women believe that their bodies are colder than their newborn baby, which may be true in some cases (34), and perhaps reinforced by the shivering that commonly follows delivery of the baby, which may greatly impact the interventions considered for warming a hypothermic baby. Newborn babies are not covered with clothes immediately after delivery, although sometimes they are wrapped with dirty clothes, preferably a *lungi* (piece of cloth wrapped around the waist) of an uncle or the mother's *saree* as the next choice. To keep a baby warm, an earthen pot containing coal is heated on a fire, then placed close to the baby to provide heat, although this is done only sporadically. Among Hindus, mothers and babies are kept together in a room isolated from others, and *dhup* (incense) is burned to emit fumes believed to expel evil spirits. Relatives rub the newborn with oil and then bathe the baby to make him/her holy (clean), even on cold nights (35; Hassan Q. Personal communication, 2001). Usually, the baby is bathed on the first day, within several hours of delivery. Among Hindus, babies are bathed after putting turmeric to the body immediately after the delivery. Only after cleaning the mother may the attendants clean the baby. Vernix caseosa is considered unholy, and attempts are made to remove it. The baby's head is shaved soon after delivery (9, 10). Foods and diseases are classified as hot (e.g. mix of cumin, chilli, and garlic) or cold, and extreme conditions are treated with food of the other type (18). Although hot foods are avoided during pregnancy, they are encouraged in the early postpartum period (16, 17). Cow's milk is seen to be a cold food and may cause the child to develop a cold or pneumonia. Therefore, it is believed that cow's milk should always be heated up. Such a precaution is not that important with *misri pani* (sugar water), another common prelacteal and supplementary food (9, 17).

#### Stillbirths

Little information is available regarding perceptions of stillbirths. Some sought hospital care, some sought traditional care, and many have contacted various health personnel of Bangladesh Rural Advancement Committee (BRAC) (23). In some reports, it was noted that stillbirths are thought to be caused by evil spirits (14–17).

### Postpartum period

As per the BDHS 2004, only 8% of mothers who did not deliver in a facility received postnatal care from a medically-trained provider (19). The most common reason given for not having a postnatal check-up was the perceived absence of need and stating that it was not customary to go. Concern about cost was an important reason. The percentage of women seeking postnatal care for their babies is also very low (18%). Less than one in five newborns is seen by a health professional within six weeks of delivery, and only 12% of babies receive a postnatal check-up by a trained health provider within the first two days of delivery (19).

One study has shown that one of the most frequently-described postpartum problems is infection, for which a wide range of local treatments is used, but very few know about antibiotics as a potential treatment for infections. Many believe in supernatural causes of disease (14).

After delivery, it is thought that the mother must remain inside the house all the time to avoid evil spirits. Mothers are strictly forbidden to go outside in the dark, in the afternoon, in a storm, after cooking, near a tubewell, with their hair down or with their *saree* (traditional dress) touching the ground—all due to fear of attracting evil spirits. It is believed that spirits live near tubewells and that the legs and hands of the mother will be bent if attacked by the spirit. Many mothers keep an iron piece close to the baby and an item of leather near the bed to ward off evil spirits. Some ask those who enter their house to wash their feet to purify the individual and protect from evil (16, 17).

#### Nutrition

**Infant feeding:** Only 28% of mothers could be considered to provide exclusive breastfeeding for five months. Most of the time, relatives (67%) provide information on breastfeeding, as do TBAs (46%) and other mothers (29%). Reasons for not breastfeeding exclusively are noted as low production of milk and poor sucking of the baby; in the latter cases, prelacteal feeds were introduced uniformly. Usually, breastfeeding is delayed until the mother is cleaned to a holy state. A practice of withholding breastfeeding up to three days after the birth of a child is also observed (32). Colostrum is rejected because it looks like pus (thick consistency) and is termed ‘dirty milk’. It is believed to be harmful (poisonous), cause diarrhoea and abdominal pain, and/or contain some evil spirit. Due to its thick and concentrated texture, it is believed that the baby would not digest colostrum. It is also considered to cause fever and illness of the mother if she feeds colostrum to the baby (9).

Introduction of prelacteal feedings is usually due to perceived delay in the flow of breastmilk, and in place of colostrum. It is also introduced to stop the baby from crying (9). As per the BDHS 2004, only 24% of children are breastfed within one hour of birth, and 87% received colostrum (19). Mostly, honey and sometimes oil (e.g. mustard oil) are used for ritual feeding. These are given to keep the baby warm, prevent fever, keep the baby's throat and stomach clear, quench thirst, and help the baby to move the mouth and, thereby, learn to eat. Sometimes, elder relatives advise the mother to give some sweet prelacteals. Palm sugar water (*misri pani* or *jol*) or water alone are also introduced by many. Sweet water or honey is given so the baby will be soft/sweet-spoken. It is also believed that a small baby can only digest sugary food (9).

Some mothers feel that, in the first 40 days of life, any other food, including cow's milk, is too cold or too strong for the baby. *Misri pani* (sweet water) is seen as having many beneficial properties. To a small child, it is food and protective medicine at the same time. For premature and low-birth-weight babies, it is seen as imperative to feed only *misri pani* along with breastmilk. Sweetness is enjoyable, and *misri pani* is believed to make a content baby sleep more. After 40 days, most who can afford it switch to cow's or goat's milk as a supplementary food, but continue to also sweeten the baby with palm sugar. The poor who cannot afford milk may give only sweetened water as a supplement until the baby is three or four months old, often with disastrous results (e.g. diarrhoea) (9, 10).

The majority of mothers also give water, often beginning at 1–3 month(s) of age. They argue that water will keep the baby well, prevent jaundice, refresh the baby, clean the blood, remove germs, and help keep the baby calm. There is not any consideration that water could be harmful for the baby during the early months of life (9).

Some do not continue breastfeeding and introduce extra food before six months, as the mothers do not think breastfeeding is enough, and they do not think it is dangerous to add these extra foods before six months of age (9). Poor nutritional status of the mother might also be a reason for termination of breastfeeding (36).

In one study, it was found that the duration of breastfeeding was shorter among mothers having secondary or higher education. Mothers from rich families had a shorter duration of breastfeeding. The reasons for this could be that affluent families could afford to buy powdered milk or baby formula. Mothers of high-income groups in urban areas tend to limit the duration of breastfeeding because they want to maintain their figures and stay attractive to their husbands, want to go back to work, or simply because they find it shameful to breastfeed (36).

There is a general belief, however, that children do not need to be weaned until they learn to walk or talk (12). Results of one study showed that birth interval positively influenced the duration of breastfeeding (36).

**Maternal nutrition:** During pregnancy and the postpartum period, especially during the first 5–9 days of seclusion, various dietary restrictions are imposed on the mother that deprive her of nutrition (see section on Antenatal period, nutrition; Table 2). Most foods, in general, are thought to be inappropriate during lactation (9, 10). For some, no food at all is allowed for the first few days after delivery, and commonly, no food is given at all during the first day after delivery to allow for healing of the birth passage (16, 17). For women suffering from severe malnutrition, this may be worsened by food taboos during the postpartum period. Women are perceived as not very hungry in the ‘house of pollution’ because they feel impure and their body stinks (9, 10). During the period of seclusion, one rice meal a day is practised in many places. The mother is advised to eat dry foods and rice with fried turmeric and onions once a day (16).

**Table 2.** Food restrictions during the postpartum period

Food restricted	Time period	To prevent
Most vegetables and vegetables with spices	1–7 day(s)	Stomach upset in both mother and newborn
Eggs, fish, and meat	One month for Hindus	Stomach upset in both mother and newborn
	One week for Muslims	
Duck eggs	Not available	Asthma in newborn
Goat meat	Not available	Allergy
Bananas, seeds, leafy vegetables, and pumpkin leaves	Not available	Not available
Beef and hilsha	Not available	Dry up a lactating mother and cause *shutika* (postpartum diarrhoea)
*Tengra* fish	Six months after delivery	Stomach pain
Guava, pineapple, coconut	Not available	Skin infection for mother and baby
*Kolmi shak*, *sheki shak,* potato (white and red)	Not available	Scabies
Some types of fresh fish	Six months for a male child	Delay in healing of umbilicus and puerperal infection
	Three months for a female child	

In many instances, the maternal diet is restricted to mashed potatoes and mashed banana for seven days after the delivery. Mashed *kalijira* (black cumin-seed) and green banana are believed to keep the stomach of a woman cool and initiate production of breastmilk. Mothers-in-law impose consumption of *kalijira* and rice for seven days after childbirth (16). Hot (spicy) food is encouraged, which is believed to heal the birth canal during the immediate postpartum period (16, 18). It is thought that if a lactating woman forces herself to eat food for which she has no appetite or desire, she could develop sutika (chronic diarrhoea in the postpartum period). In doing so, she may cause problems for the next child she carries, affecting negatively his/her health or character (9).

Women eat fish, especially *magur*, *shing*, and *koi* to increase the production of breastmilk. It is always on the menu of special ritual meals fed to women to celebrate the birth. Milk, eggs, poultry, and lentils are also seen as highly nutritious. To the extent possible, these, together with fish, are prepared once in a special ritual meal offered to the postpartum mother to ensure that she regains her strength and produces plenty of breastmilk. However, the celebration is cancelled if the baby dies in the meantime, suggesting that the purpose is not so much to restore the mother's strength as to stimulate production of breastmilk and celebrate the new life of the child (9, 10). *Bailla*, *coral* and *tengra* fish, and dried fish with chillies are believed to heal the birth canal, post-delivery infection, and the umbilicus of the baby. These are believed to protect mothers from pain (16).

#### Danger signs

Postpartum bleeding is not a matter of concern unless it gets very heavy. The elimination of a certain quantity of blood is accepted and seen as beneficial to the mother, as blood coming from the womb is considered polluted and harmful, and best dispelled. A woman who does not bleed sufficiently is thought to have developed complications and is subject to a slower recovery. Thus, postpartum haemorrhage may not be recognized until too late when the mother's life is already in jeopardy (9, 10). On the other hand, it is also believed that the mother will turn into a mad person if excessive postpartum bleeding occurs (16).

### Care-seeking behaviour

#### Decision-making

In general, fathers are less knowledgeable than mothers regarding pregnancy and delivery-related issues and are reluctant to take responsibility for decisions. Mothers make decisions regarding infant and child health, e.g. care of respiratory infections, diarrhoeal diseases, immunizations, (36), while the father, paternal grandmother, and other relatives make decisions regarding seeking care outside the home. Care-seeking behaviour for complications during delivery was noted to depend on the economic situation of the family. Care-seeking from trained providers was found to be associated with education of the father, and fathers generally took decisions for seeking care outside the home from a trained provider. Families with a monthly expenditure of Tk 4,000 or greater were more likely to seek care for their newborns than families with a monthly expenditure of less than Tk 2,000 (14, 15).

In a comparative study conducted of BRAC beneficiaries (i.e. members of BRAC's integrated Rural Development Programmes) and non-beneficiaries, it was found that gender differentials in the context of health-seeking behaviour persisted among the beneficiaries despite participation in the programme. Women sought treatment significantly less often than men, reflecting the strong influence of patriarchy in rural Bangladesh society. Also, for a male child and the first born, decisions to seek outside help are prioritized (27).

#### Choice of provider

In a survey of urban slum women (n=122), two-thirds reported non-trivial illness. Despite poor socioeconomic circumstances, 71% of them sought some form of healthcare, although generally from unqualified traditional practitioners (37). Of note, BRAC beneficiaries use more home-remedies, and traditional care and seek unqualified allopaths more than non-BRAC beneficiaries who have more tendency to use para-professional services available in the community and to consult healthcare workers (24). Most rural population favour village doctors, as they are cheaper and accessible, and their practices are considered culturally acceptable (9, 14, 15). In one study, 87.5% of women went to a village doctor, 48.5% to Upazila Health Complex (UHC), 44% to homeopaths, 35% to medical doctors/clinics, 35% to traditional healers, 20% to a pharmacy, and only 4% to Medical Assistants in more peripheral health facilities (14). Although the governmental health facilities are free, this use is poor. Often, the patient will be referred to the UHC or district hospitals only as a last resort (9, 10, 14, 15).

In general, the custom of seclusion after delivery is a key obstacle to healthcare-seeking behaviour. Regarding the sources of care for intrapartum complications in rural areas, as with care-seeking for general health issues, the provider who is approached most commonly is a village doctor, followed by doctors/nurses or governmental health facilities, homeopaths, TBAs, and, for some conditions, pharmacies (15).

Table 3 summarizes some reasons given for not seeking healthcare. Seeking care from traditional healers and lay-persons versus seeking care from the formal health sector depends on a number of factors, including income, education, exposure to modern health services, and quality and cost of services (9, 10). Reasons for not attending hospitals include poor quality of hospital services and behaviour of hospital staff, difficulties of transport, fear of being operated on, cost, misconception of treatment, and requirements for bribes (16, 17). Reasons for preferring deliveries at home include: relatives at home provide mental support, which hospital lacks; elder relatives assure the mother ‘we are for you at your crisis’; privacy is available; and it is customary, as the vast majority of deliveries are conducted at home with acceptable outcomes (14, 17).

**Table 3.** Reasons for not seeking healthcare duirng the postpartum period

Physical insecurities	Distance of the hospital from the community and lack of transportation
	Seclusion of the mother after delivery, prevented from travelling alone
	Lack of access to maternal and child health services
Social insecurities	Sex of baby
	Lack of support from family (husbands, mothers, mothers-in-law)
	Low decision-making power within household
	Lack of or low education of father
	Use of alternative options for medical care: village doctors, homeopaths, traditional healers, etc.
	Fear of dishonour due to exposure to men, examination by a male doctor
Economic insecurities	Lack of funds
	Requirement of bribes
Environmental insecurities	Unfriendly service at facilities
	Culturally-inappropriate methods and medications used at facilities
	Fear of being operated on and no prior exposure to modern health facilities
	Lack of privacy

#### Care seeking for neonatal illness

One study in Bangladesh (36) reported that 87% of mothers sought care for their neonates who had problems (fever, breathing problems and cold, or upper respiratory tract illness) (9, 10). Only 17% were taken to trained providers, whereas 38% were taken to homeopaths, and 37% were taken to village doctors. Homeopathic medicines are believed to be mild, slow in action with no side-effects, and especially suitable for children, particularly newborns, because of their sweet taste and ease of administration. Doctors tended to be consulted for particular problems, such as umbilical redness or discharge, diarrhoea, breathing problems, and loss of weight (8).

## DISCUSSION

Existing literature on neonatal care in Bangladesh does not cover many important issues. The raison d’être of the SNLI is based on the realization that very little has been done in this area, and very little attention has been paid to the subject of the newborn, particularly the impact that domiciliary practices have on neonatal health.

Based on the sheer number of practices/behaviours performed in the course of routine and sick newborn care and the difficulty in changing behaviours that may be firmly entrenched through generations of ritual belief and practice, is essential to prioritize those behaviours which are amenable to change, and which, if modified, would be expected to have the greatest impact on newborn health and survival.

An evaluation of the existing knowledge, attitudes, and practices regarding neonatal care provided in the home by caretakers, community health workers, *dais*, and trained birth attendants, including a clear understanding of health-seeking behaviour for neonatal illness, would greatly inform the design of effective prevention and treatment strategies to improve newborn health at the community levels (38). Studies have shown that antenatal and postnatal care can be considerably improved through implementing interventions at the family and community levels, including health education to improve domiciliary neonatal—care practices and health-seeking behaviour for neonatal illness (5).

Addressing home-based care for the mother and her newborn has not been incorporated widely into public-health programmes or monitored by the demographic and health service surveys (5). Formative research in this area could be used for helping design and develop various programme activities, including *dais* and TBAs, educating mothers and other caretakers, creating demand for skilled care by improving health-seeking behaviour, increasing referral rates, and designing a package of simple and culturally-acceptable practices for routine postpartum care of neonates, which would include proper thermal control, promotion of early and exclusive breastfeeding, and optimal skin and hygienic cord care (7).

Based on the results of this review of the literature on community-based maternal and newborn practices, a number of priority areas/behaviours for further formative research were identified (Table 4). Stemming from this review, in combination with a critical evaluation of evidence for impact of antenatal, intrapartum and postpartum interventions/behaviours on perinatal and neonatal outcomes (39), a guide to formative research on community-based newborn-care practices has been developed by the SNLI (7). The information gained from this review of the literature, supplemented with data gained through additional formative research conducted on priority newborn-care behaviours will form the foundation for, and will greatly facilitate, the development of an effective behaviour change-communications strategy for the advancement of neonatal health and survival in Bangladesh.

**Table 4.** Priority antenatal, intrapartum, and postpartum behaviours/care for further formative research

Antenatal
Reasons for lack of emphasis on consumption of iron/folate supplement
Knowledge/perceptions of sexually transmitted/reproductive tract infections: risk factors and modes for acquisition and transmission, consequences for health of mother and baby
Overcoming barriers to antenatal care attendance
Perceptions and impact of smoke/environmental pollutants (e.g. indoor, outdoor air pollution, smoking) and other substance abuse
Overcoming barriers to birth-preparedness
Overcoming barriers to care-seeking for pregnancy-related complications
Intrapartum/immediate newborn care
Perceptions of hygiene/cleanliness and risk factors for infections, particularly with regard to clean delivery and clean cord care
Modification of harmful practices to speed delivery of baby and/or placenta
Overcoming delays in initiation of newborn resuscitation
Overcoming barriers to effective resuscitation of non-breathing baby
Overcoming delays in drying and warming newborn immediately after birth
Perceptions regarding stillbirths
Postpartum
Overcoming barrier to routine postpartum care for mother and baby
Perceptions regarding prelacteal and supplementary feeding
Recognition of maternal and newborn danger signs
Overcoming delays to care-seeking for illness

## ACKNOWLEDGEMENTS

Save the Children-USA supported the study through a grant from the Bill and Melinda Gates Foundation.
